# Quantitative CMR markers of impaired vascular reactivity associated with age and peripheral artery disease

**DOI:** 10.1186/1532-429X-15-17

**Published:** 2013-02-13

**Authors:** Michael C Langham, Erin K Englund, Emile R Mohler, Cheng Li, Zachary B Rodgers, Thomas F Floyd, Felix W Wehrli

**Affiliations:** 1 Department of Radiology, University of Pennsylvania Medical Center, 3400 Spruce Street, Philadelphia, PA, 19104, USA; 2 Department of Medicine, University of Pennsylvania School of Medicine, Philadelphia, PA, 19104, USA; 3 Department of Anesthesiology, Stony Brook University Medical Center, Stony Brook, NY, 11794, USA

**Keywords:** Peripheral arterial disease, Atherosclerosis, Microvascular function, Pulse-wave velocity, Blood oxygen saturation, Phase image, Magnetic resonance oximetry

## Abstract

**Background:**

The aim of this study was to develop and evaluate an integrated CMR method incorporating dynamic oximetry, blood flow and pulse-wave velocimetry to assess vascular reactivity in patients with peripheral artery disease (PAD) and healthy controls.

**Methods and results:**

The study population consisted of young healthy subjects (YH, 30 ± 7 yrs, N = 19),PAD (71 ± 9 yrs, N = 38), and older healthy controls (OHC, 68 ± 9 yrs, N = 43). Peripheral vascular reactivity was evaluated with two methods, time-resolved quantification of blood flow velocity and oxygenation level in the femoral artery and vein, respectively, performed simultaneously both at rest and hyperemia. Aortic stiffness was assessed via pulse-wave velocity. Oximetric data showed that compared to OHC, the time-course of the hemoglobin oxygen saturation in PAD patients had longer washout time (28.6 ± 1.2 vs 16.9 ± 1.1 s, p < 0.0001), reduced upslope (0.60 ± 0.1 vs 1.3 ± 0.08 HbO_2_/sec, p < 0.0001) and lower overshoot (8 ± 1.4 vs 14 ± 1.2 HbO_2_, p = 0.0064). PAD patients also had longer-lasting antegrade femoral artery flow during hyperemia (51 ± 2.1 vs 24 ± 1.8 s, p < 0.0001), and reduced peak-to-baseline flow rate (3.1 ± 0.5 vs 7.4 ± 0.4, p < 0.0001). Further, the pulsatility at rest was reduced (0.75 ± 0.32 vs 5.2 ± 0.3, p < 0.0001), and aortic PWV was elevated (10.2 ± 0.4 vs 8.1 ± 0.4 m/s, p = 0.0048).

**Conclusion:**

The proposed CMR protocol quantifies multiple aspects of vascular reactivity and represents an initial step toward development of a potential tool for evaluating interventions, extrapolating clinical outcomes and predicting functional endpoints based on quantitative parameters.

## Background

Peripheral artery disease (PAD) is most commonly due to atherosclerosis. Currently, it is estimated that approximately ten million people in the United States are affected by PAD and the number is expected to grow as the population ages [1]. In spite of its high prevalence the disease often goes unnoticed because the vast majority of PAD patients have no classic claudication symptoms, which typically occur at an advanced stage. The initial test for diagnosing patients with clinical symptoms is measurement of the ankle-brachial index (ABI). A low ABI is a strong indicator of the presence of PAD but a normal ABI does not rule out risk due to the false negative rates [2], which can be understood based on the fact that the pressure decrease in the ankle can only be detected when lesions reduce the lumen’s diameter by ≥ 70%.

Some of the manifestations of structural and functional alterations that associated with PAD involve increases in intimal-medial vessel wall thickness (IMT) [3], decreased vascular compliance [4], and impaired vascular reactivity [5]. Flow-mediated dilation (FMD) of the brachial artery following cuff-induced ischemia [6] is a widely used research technique for quantifying vascular reactivity. B-mode ultrasound measures intimal-medial thickness (IMT), typically performed in the common carotid artery, a procedure practiced clinically for cardiovascular disease (CVD) risk assessment [7,8]. Major limitations of ultrasound are poor reproducibility due to high inter- and intra-observer variability [9,10]. Further, the above US measures do not target vascular territories (brachial and carotid arteries) that are most commonly affected by PAD (lower peripheral arteries). Increased arterial stiffness is also associated with atherosclerosis [11-13] and may contribute to microvascular dysfunction [14]. Aortic stiffness can be estimated by quantifying PWV, typically by measuring the time delay of the systolic pressure wave at some downstream location, using pressure transducers [15,16] placed at the two locations, e.g. carotid and common femoral arteries. However, carotid-femoral PWV is merely a surrogate [17] for aortic arch PWV because by the time the pressure wave is detected at the carotid artery it has already propagated through the ascending aorta.

Other non-invasive imaging techniques for quantifying reactive hyperemia in peripheral arteries include near-infrared spectroscopy (NIRS) [18-21], single photon emission computed tomography [22] and CMR [23-25]. The NIRS is also a widely used research tool due to portability and low cost. It has excellent temporal resolution, is less prone to artifacts from subject motion, and allows simultaneous monitoring of relative changes in tissue blood flow and oxygenation. However, the spatial resolution of NIRS is limited and it can only target the capillary bed in relatively superficial tissues. Further, the modality is susceptible to large inter-subject variations in the tissue response to light, which depends on skin color, body fat and muscle layers. CMR flow velocimetry [23,24] has been shown to provide high spatial and temporal resolution during post-occlusion hyperemia in the femoral artery of PAD patients and healthy subjects at 1.5T. The method requires spatially-selective RF pulses which are not widely available and migration to systems with higher field strength may be challenging due to increased field in homogeneity. Alternatively, microvascular function can be assessed by quantifying post-occlusive perfusion with arterial spin labeling (ASL) in calf muscle [26]. The ASL-approach is model-dependent, and the temporal and spatial resolutions are limited.

Here, we designed, implemented and evaluated an integrated CMR protocol that captures quantitative markers of vascular reactivity in the lower extremity and the aortic arch in a single examination. The markers include parameters derived from post-occlusive blood flow velocity and oxygenation levels (HbO_2_) in the femoral vessels at high temporal resolution, and pulse-wave velocity for assessing the stiffness of the aortic arch. The method represents a significant improvement from the authors’ pilot study [27] where only HbO_2_ was monitored during reactive hyperemia to assess peripheral vascular reactivity. The present work also represents an initial step toward development of a tool for extrapolating clinical outcomes and predicting functional endpoints based on quantitative parameters.

## Methods

### Study participants

Three groups of subjects were studied: patients with PAD (PAD; N = 38, mean ABI 0.69±0.15, mean age 64.6±7.7 yrs, 14 females), older healthy controls (OHC; N = 43, mean ABI 1.12±0.09, mean age 61± 7.6 yrs, 25 females) and young healthy subjects (YH; N = 19, mean ABI 1.1±0.12, mean age 34.3±7.5 yrs, 8 females). Additional patient information is summarized in Table 1. YH and OHC were recruited based on their ABI (>0.90 in either leg) and medical history (normotensive and without prior cardiovascular events). Patients included men and women with a history of PAD, defined as having ABI < 0.90. Patients with a history of myocardial infarction or stroke within three months prior to study enrollment, or vascular surgery on the leg selected for scanning were excluded. Written informed consent was obtained prior to all examinations following an institutional review board-approved protocol.

**Table 1:** Subject characteristics; means and standard deviations (in parenthesis)

	**OHC**	**PAD**	**YH**
**N**	**43**	**38**	**19**
Age, yrs	61 (7.6)	64.6 (7.7)	34.3* (7.5)
ABI	1.12 (0.09)	0.69* (0.15)	1.1 (0.12)
BMI, kg/m^2^	23 (3.0)	28* (5)	24 (4)
Systolic BP, mmHg	122 (16)	139* (26)	114 (15)
Diastolic BP, mmHg	76 (10)	80 (11)	75 (10)
MAP, mmHg	91 (10)	100* (15)	88 (11)
Heart rate, beats/min	67 (10)	73 (16)	71 (7)
HDL, mg/dL	58* (19)	46 (11)	44 (11)
Tot. Cholesterol, mg/dL	204* (36)	169 (46)	173 (26)

Asterisk indicates significant different (p < 0.0167) compared to other groups.

### Integrated CMR assessment of vascular reactivity

The inclusion of aortic PWV quantification is motivated by the fact that abnormal aortic stiffness can lead to increased transmission of pressure pulsatility to peripheral vessels that can affect microvascular reactivity leading to ischemic stress [14]. However, regional PWV of the thoraco-abdominal aorta and from iliac to femoral arteries would be more relevant since the gradient of PWV at the aorto-iliac bifurcation affects impedance matching which, in turn, determines the degree of transmission of pressure waves to peripheral vessels. All CMR studies were performed at 3T field strength (Siemens TIM Trio, Siemens Medical Solutions). The femoral artery and vein were imaged with an eight-channel phased-array knee coil (In vivo Inc., Pewaukee, WI) and the manufacturer’s body matrix coil (with chest elements activated) was used to image the aorta.

#### Quantification of reactive hyperemia in response to cuff-induced ischemia

Post-occlusive reactive hyperemia was measured from the time-course of blood oxygen saturation (HbO_2_) in the femoral vein [27] and femoral artery flow velocity [28]. Lower-leg ischemia was induced with a blood-pressure cuff (Aspen Labs A.T.S. 1500 Tourniquet System, Littleton, CO) applied to the upper thigh at 75 mm Hg above the subject’s systolic blood pressure but not exceeding 250 mmHg. The cuff paradigm consisted of 2 mins baseline, 5 mins of occlusion and 6 mins of recovery. During baseline (i.e. before cuff inflation) blood flow velocity in the femoral artery and HbO_2_ in the femoral vein were measured. Blood flow velocity was measured for eight to ten cardiac cycles at a temporal resolution of 20 ms, followed by three measurements of HbO_2_. Data collection was suspended during cuff occlusion and resumed ten seconds prior to cuff deflation. Both quantities were then measured simultaneously in an interleaved fashion for 80 s while for the remaining recovery period (four minutes and fifty seconds) only HbO_2_ was monitored.

#### Measurement of arterial blood flow velocity

Blood flow velocity in the femoral artery was time-resolved without cardiac gating during baseline and hyperemia by collecting velocity-encoded projections [28] resulting in 40-50 time points sampled within each cardiac cycle. Imaging parameters for baseline blood flow velocity quantification: TE/TR = 5.23/10 ms, flip angle = 15°, bandwidth = 520 Hz/pix, voxel size = 1 × 5 mm^2^, matrix size = 128 and VENC = 60 cm/s (user defined parameter that defines the maximum measurable velocity).

#### Measurement of HbO2

HbO_2_ in the femoral vein was estimated by field mapping, which yields the susceptibility difference Δχ between intravascular blood and surrounding tissue [29], where the Δχ is proportional to (1-HbO_2_) and hematocrit (determined from a blood sample). Imaging parameters used for baseline HbO_2_ quantification were: TE/TR =4.3/156.25 ms, flip angle = 25°, bandwidth = 520 Hz/pix, voxel size = 1 × 1 × 5 mm^3^, matrix size = 128 × 128, yielding a temporal resolution of 20 s during baseline.

#### Simultaneous quantification of HbO_2_ and blood flow velocity during hyperemia

Ten seconds prior to the cuff deflation an interleaved multi-echo GRE pulse sequence [30] was launched to collect velocity-encoded projections (for velocity measurement) in addition to full images (for venous HbO2 measurement). Imaging parameters during hyperemia were: TE/TR = 5.0/39.1 ms, flip angle = 15°, bandwidth = 520 Hz/pix voxel size = 1 × 1 × 5 mm^3^, matrix size = 128 × 128. Velocity encoding (VENC) parameters were 125cm/s (YH and OHC) and 80 cm/s for PADs, the rationale being that flow in the patients would be lower than in healthy subjects. The interleaved pulse sequence HbO_2_ and blood flow velocity simultaneously during reactive hyperemia with temporal resolution of 5 s and 120 ms, respectively.

#### Aortic arch pulse-wave velocity

**Figure 1:**
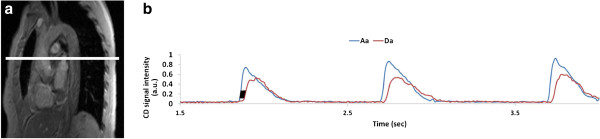
**Scout images for quantifying aortic arch PWV. a**) Oblique sagittal image of the aorta for prescribing an axial image; **b**) Complex difference signal intensity plotted as a function of time showing approximately three cardiac cycles. The transit time of the pressure pulse wave is estimated by computing the average “width” of the black parallelogram (first full cardiac cycle around t = 2 s). The height of the parallelogram was prescribed using a graphical user interface written in C++.

**Figure 2:**
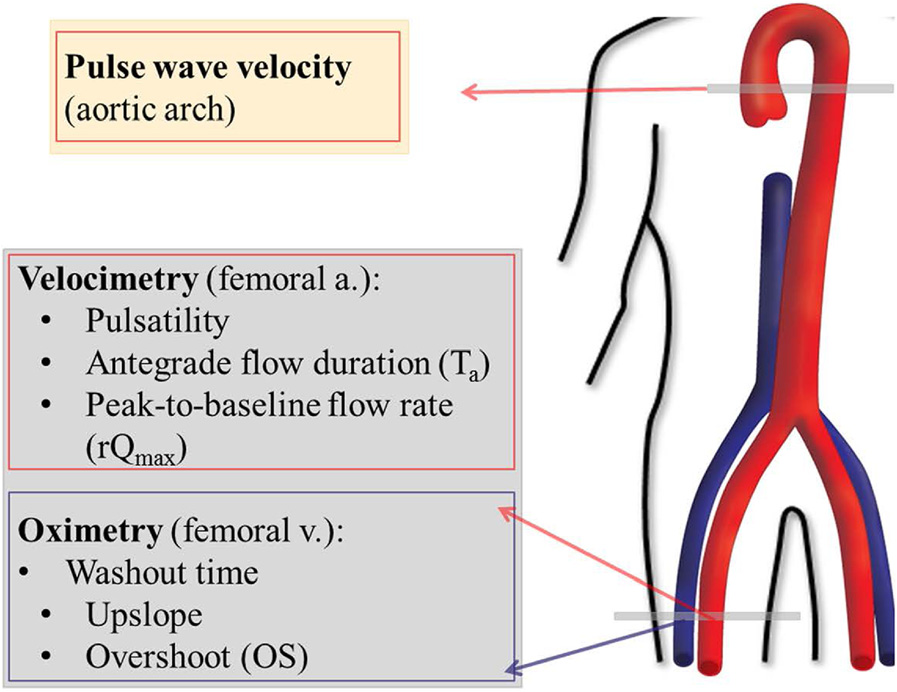
**The anatomical locations and the measured parameters of the integrated MRI protocol for evaluating vascular reactivity.** See Data Analysis for the definition of the parameters.

Following the extremity protocol the patient was prepared for aortic arch PWV quantification. The manufacturer’s standard body matrix coil was positioned on the chest and multiple axial slices below the pulmonary trunk were obtained from an oblique sagittal image of the aorta (Figure 1a) to select an axial slice for simultaneously visualizing ascending and proximal descending aorta. Analogous to the measurement of arterial blood flow velocity, velocity-encoded projections were obtained. The time-course of the complex difference (CD) signal intensity (Figure 1b) of velocity-encoded projection signals is approximately proportional to the velocity for large velocity encoding. The time-point of onset of rise in systolic arterial velocity is delayed downstream, yielding the wave-front propagation time [31]. Imaging was performed with the following parameters: FOV = 448 mm, voxel size = 2 × 10 mm^2^, TE/TR = 2.6/5.0 ms, bandwidth = 893 Hz/pixel, flip angle = 15° and *VENC* = 175 cm/s. After collecting a reference image (for the purpose of identifying the vessels in CD images) 1024 pairs of velocity-encoded projections were acquired in free-breathing mode, covering about 10–12 heart-beats at a temporal resolution of 10 ms. The data acquisition time was approximately 12 s. A summary of the integrated MRI protocol is shown in Figure 2 highlighting the anatomic locations of the three procedures and the parameters derived.

### Data analysis

#### Time-resolved blood flow velocity

**Figure 3:**
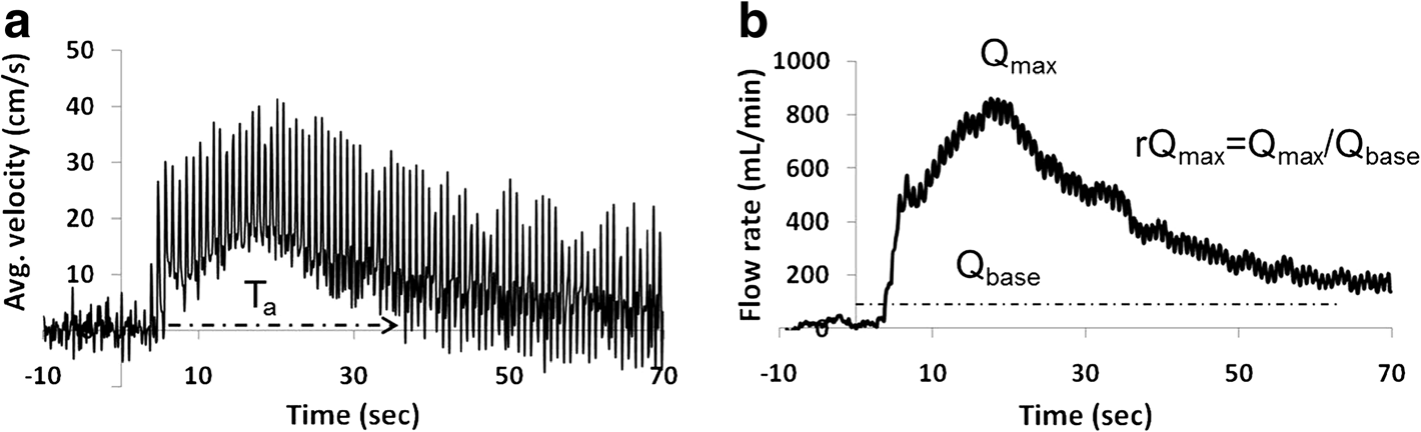
**a) Time-resolved post-occlusive blood flow velocity in the femoral artery of a healthy young subject.** The cuff was released at *t* = 0 and each vertical “spike” represents a systolic peak. The time T_a_ (arrow) represents the duration of the forward flow during hyperemia due to reduction in the vascular resistance. **b**) Flow rate computed from the velocity data averaged over a 2-second sliding window. The dashed line represents the baseline flow rate.

The phase differences between velocity-encoded projections (after removal of tissue signal) were converted to velocity using the well-known relation between phase *φ* and velocity *v* yielded average peak-systolic velocity *v*_*s*_, peak-retrograde velocity *v*_*r*_ and peak late-ante grade velocity *v*_*a*_. From these parameters a measure of pulsatility, defined as (*v*_*s*_*- v*_*r*_)/*v*_*avg*_, was computed. The time-course of the hyperemic blood flow velocity (Figure 3a) was expressed in terms of the *duration of ante grade flow, *T_a_, and *peak-to-baseline flow rate* (rQ_max_) (Figure 3b), defined as the ratio, *v*_*peak,avg*_*/v*_*base,avg*_. The parameter *v*_*base,avg*_ was computed by averaging the time-resolved velocity during baseline over 6–8 cardiac cycles and *v*_*peak,avg*_ is the peak of the temporally averaged velocity via a 2-second sliding window during the course of hyperemia.

#### Time-resolved HbO_2_ quantification

**Figure 4:**
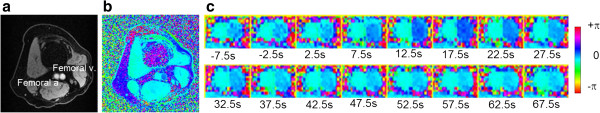
**Representative magnitude (a) and phase difference image (b). c)** Magnified views of the dynamic changes in the phase of femoral vein blood captured with dynamic oximetry. Image frames are displayed for a period ranging from t = −7.5 to 67.5 s, at a temporal resolution of 5 s. Note, the sixth frame corresponds to the minimum venous saturation. Note rapid change in HbO_2_ during passage of desaturated blood.

After appropriate preprocessing of the phase images [32], HbO2 was computed from phase difference between intra- and extravascular compartments as described in [29,33]. Figure 4 shows representative magnitude and phase difference images containing femoral artery and vein, and the magnified phase difference images of the vessels during hyperemia.

**Figure 5:**
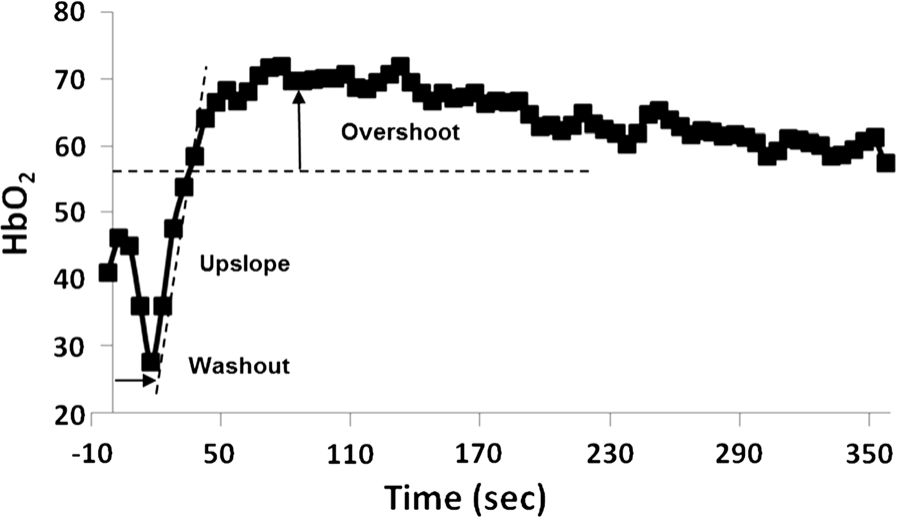
**Temporal changes in HbO**_**2 **_**and parameters quantified from dynamic oximetry.** Cuff deflation occurs at t = 0 (vertical axis).

The time-course of *HbO*_*2*_ (Figure 5) characterizes vascular reactivity expressed in terms of three parameters *washout time*, *upslope* and *overshoot*, as described previously [27]. The washout time is the time elapsed to observe the oxygen-depleted capillary blood at the imaging slice after cuff deflation that occurs at t = 0. *Upslope* and *overshoot* are defined as the mean resaturation rate during hyperemia and the subsequent above-baseline *HbO*_*2*_ level, respectively.

#### Pulse-wave velocity

The CD signals from ascending and proximal descending aorta were spatially averaged along the readout direction within the vessel boundary for each time point and are plotted jointly to estimate the propagation time of the wave-front (commonly known as the “foot-to-foot” method) [31] (Figure 1b). The estimation of the wave-front propagation time illustrated in Figure 1b is equivalent to the foot-to-foot technique commonly utilized in tonometric studies [34]. This measurement was performed for each heart beat and averaged over the 8 to 10 cardiac cycles. The path length *L* of the wave-front was then estimated from a center-line of the aorta between the two aortic sites manually drawn on an oblique sagittal image of the aortic arch and PWV is calculated as, $L/\bar{\mathrm{\Delta t}}$, where $\bar{\mathrm{\Delta t}}$ is the propagation time averaged over multiple heart-beats.

### Statistical analysis

Non-parametric multiple comparisons (JMP 9.0 SAS Institute Inc.) were performed for each of the seven parameters using a Wilcoxon test. An association was considered significant when p < 0.0167, determined by applying a Bonferroni correction to account for multiple comparisons.

## Results

**Figure 6:**
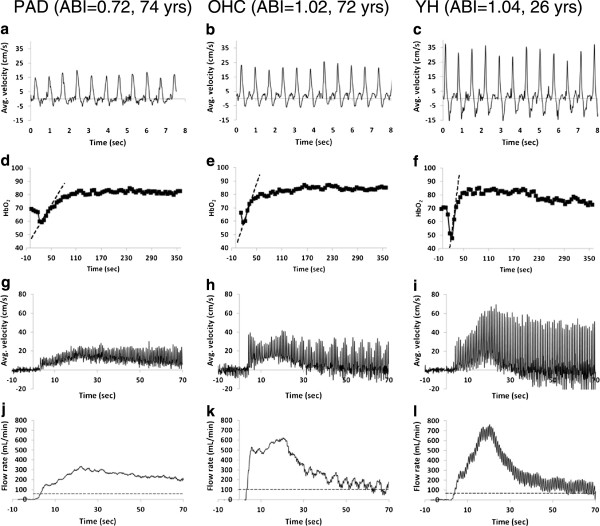
**Each column of panels shows time-courses of baseline blood flow velocity (a – c), HbO**_**2 **_**(d – f) hyperemic arterial velocity (d – f) and flow rate (j – l) in a patient with PAD without intermittent claudication (ABI = 0.72, age 74 yrs, a, d, g, j), OHC (ABI = 1.02, age 72 yrs, b, e, h, k) and YH (ABI = 1.04, age 26 yrs, c, f, i, l), respectively.** The dashed lines in **d**-**f** and **j**-**l** represent upslope and baseline flow rate, respectively.

In Figure 6 baseline blood flow velocity wave form and time-courses of post-occlusion HbO_2_, blood flow velocity and flow rate are shown of a patient with PAD and compared with the corresponding data from two healthy subjects (OHC and YH). The data illustrate the differences in peripheral vascular reactivity between three cohorts. Specifically, the velocity components of retrograde and late-ante grade flow are better defined in OHC compared to the patient (Figure 6a and b). The HbO_2_ time-course indicates a prolonged washout time and reduced upslope in the PAD patient compared to the OHC (Figure 6d and e). In the post-occlusion velocity time-course (Figure 6g, h), the peak average velocity of the PAD patient is significantly attenuated (resulting smaller peak flow rate as shown in Figure 6j, k) compared to the OHC, an effect that appears to be compensated by a prolonged recovery period [35]. In Figure 6, the age difference between the OHC and YH is about 45 yrs and the difference in “vascular age” is characterized by a reduction in pulsatility (Figure 6b, c), upslope, overshoot (Figure 6e, f) and maximum relative increase in the flow rate (Figure 6k, l).

**Figure 7:**
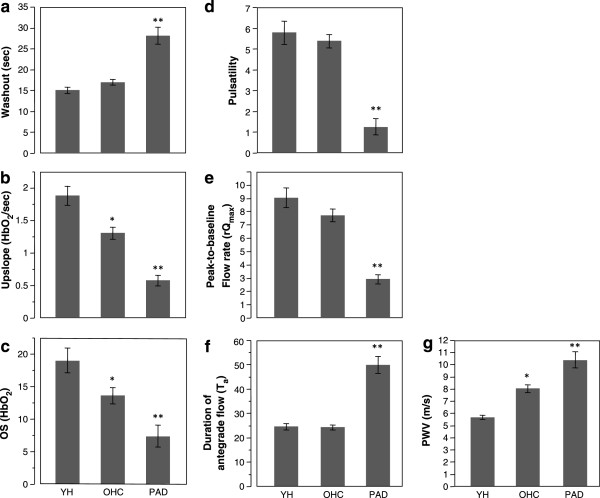
**Average values of the parameters of (a-c) oximetry, (d-f) velocimetry and g) aortic PWV.** Error bars represent standard error: single asterisks refer to the mean difference in parameters between OHC and YH: upslope (p < 0.005), OS (p = 0.016) and PWV (p < 0.001). Similarly, double asterisks refer to the mean difference in parameters between OHC and PAD: all p < 0.0001 except for OS (p < 0.001) and PWV (p < 0.005).

**Figure 8:**
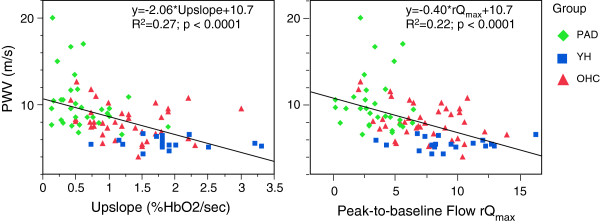
**Correlation between aortic PWV and microvascular reactivity.** The parameters with the strongest correlation with PWV were upslope and rQ_max_.

Results for the seven parameters are shown in Figure 7 for each group and average values and standard errors for each parameter and groups are summarized in Table 2. In accordance with the data from three representative subjects in Figure 6 the HbO_2_ time-courses of PAD patients indicated longer washout time (28.6 ± 1.2 vs 16.9 ± 1.1 s, p < 0.0001), lower upslope (0.60 ± 0.1 vs 1.3 ± 0.08 HbO_2_/sec, p < 0.0001) and overshoot (8 ± 1.4 vs. 14 ± 1.2HbO_2_, p < 0.0064) compared to OHC (Figure 7a-c). The low pulsatility index (Figure 7d, 0.75 ± 0.32 vs 5.2 ± 0.3, p < 0.0001) of PAD compared to OHC results from reduced peak systolic velocity and attenuation of retrograde flow, as illustrated in Figure 6a. Further, the reduced average velocity during hyperemia in PAD patients translates into smaller peak-to-baseline flow rate (Figure 7e, 3.1 ± 0.5 vs 7.4 ± 0.4, p < 0.0001) while increasing the duration of the post-occlusion antegrade flow (Figure 7f, 51 ± 2.1 vs 24 ± 1.8 s, p < 0.0001). The aortic arch PWV was elevated (Figure 7g, 10.2 ± 0.4 vs 8.1 ± 0.4 m/s, p = 0.0048) in patients compared to OHC. Among the healthy subjects (YH and OHC) greater upslope (Figure 7b, 1.88 ± 0.13 vs. 1.3 ± 0.08HbO_2_/s, p = 0.0019), overshoot (Figure 7c, 19 ± 1.9 vs. 14 ± 1.3 HbO_2_, p = 0.016), and as expected, smaller PWV (Figure 7g, 5.6 ± 0.5 vs. 8.1 ± 0.4 m/s, p < 0.001) were observed in YH compared to OHC. The correlation between stiffening of central artery and decline in peripheral vascular reactivity is demonstrated by the inverse relationship between PWV and both upslope and rQ_max_ (Figure 8a and b, respectively).

**Table 2:** Average (standard error) of parameters of vascular reactivity

**Groups**	**Washout (s)**	**Upslope (HbO**_**2**_**/s)**	**Overshoot (HbO**_**2**_**)**	**Pulsatility**	**T**_**a**_**(s)**	**rQ**_**max**_	**PWV (m/s)**
OHC	16.9 (1.1)	1.3 (0.08)	14 (1.2)	5.2 (0.3)	24 (1.8)	7.4 (0.4)	8.1 (0.4)
PAD	28.6 (1.2)	0.60 (0.1)	8 (1.4)	0.75 (0.32)	51 (2.1)	3.1 (0.5)	10.2 (0.4)
YH	15 (1.7)	1.88 (0.13)	19 (1.9)	5.76 (0.42)	25 (2.6)	9.0 (0.6)	5.6 (0.5)

## Discussion and conclusion

In this work we have presented an integrated CMR protocol that is able to quantify multiple physiological parameters associated with vascular reactivity and systemic compliance in patients with established PAD and healthy control subjects. The data indicate that highly significant differences in the measured parameters between PAD patients and OHC, as well as between the latter and the YH (Figure 7 and Table 2). Longer washout time and increased duration of antegrade flow, T_a_, reduced upslope, overshoot, pulsatility and rQ_max_, as well as elevated aortic arch PWV, are suggestive of impaired vascular reactivity in the PAD patients relative to age-matched control subjects. The underlying rationale is that during cuff occlusion the arterial supply is disrupted in the lower extremities, thereby leading to gradual depletion of tissue oxygen stores. Thus, the washout time and upslope characterize the rate at which blood in hypoxic tissue is replaced, which is a function of the reactivity of the microvasculature since the latter is the primary source of vascular resistance. The transient increase in arterial blood flow during hyperemia provides further insight into vascular system’s ability to respond to an ischemic stimulus. The brief absence of flow reversal in healthy subjects during hyperemia (Figure 6h, i) is likely the result of the reduction in microvascular resistance, which can accommodate seven- to nine-fold transitory increase in the flow rate [36].

Our results of aortic PWV (PAD vs. OHC) support previous studies that have shown a correlation between elevated aortic PWV and atherosclerosis [37,38] as well as microvascular dysfunction [14], as shown in Figure 8. Mitchell et al. [14] also observed that the correlation persisted even in models that adjusted for shared risk factors. Approximately 50% of the PAD patients were on beta-blocker therapy (most on metoprolol) and the average systolic blood pressure (SBP) of the patient cohort indicated pre-hypertension (139 ± 26 mmHg), but the SBP was higher than that of the OHC subjects (122 ± 16 mmHg, p = 0.02). Thus, it could be argued that the increase in the aortic PWV among PAD patients may be the result of elevated SBP, which is common [39,40] among PAD patients. However, we did not find a correlation between elevated SBP and aortic PWV in PAD patients and OHC subjects as a whole. Further, all PAD patients were on some class of anti-hypertensive medication (ACE inhibitor, beta blocker, diuretics and calcium channel blocker) that are, in general, known to be beneficial in reducing arterial stiffness [41]. The known beta blockers that increase PWV are propanolol, bisoprolol and possibly atenolol. In our patient cohort, only four subjects were on atenolol and one on propanolol. Thus, we think the medications had minimal impact on the observed difference in the PWV between PAD and OHC. Nevertheless, our data suggest that aortic PWV has the potential to complement the parameters of reactive hyperemia for assessing PAD, which is in agreement with the notion that excessive transmission of pulse pressure can have deleterious effect on microvascular reactivity [14]. Lastly, MRI-based PWV quantification, which had been validated against invasive pressure measurements [42], is more versatile as it is able to provide regional PWV [43] with higher accuracy [25,42] compared to the transducer-based method [44], which is merely a surrogate marker of aortic PWV [17].

From the results of the oximetric parameters we further observed an age-related decline in vascular reactivity. Among healthy subjects the upslope and overshoot were significantly greater in YH compared to OHC subjects (Figure 7a, c).

There are some limitations of the techniques utilized in the present study. The application of the cuff resulted in exclusion of patients with stent implants in the femoral or popliteal artery. Thus, it was not be possible to evaluate vascular reactivity following stent intervention. Further, the quantification of blood flow and oxygen saturation in the conduit artery and vein provides only a global assessment (i.e. yielding no information on specific vascular territories affected in advanced disease, as opposed to magnetic resonance angiography, for example). The method therefore does not provide information on tissue perfusion of specific muscle groups where an oxygen deficit may be present. In general, the reduced blood flow velocity and increased duration of antegrade flow also depend on the effects of upstream stenosis (especially PAD patients) and not just on the downstream microvascular dysfunction. Thus the velocity data of PAD may represent the combined effects of macro- and microvascular deficits. Also, it should be noted that our method may underestimate rQ_max_ values by 3 to 5% since we ignored the very small change in lumen area that is not detectable at the resolution of 1mm. PWV was quantified from velocity-encoded projections that resolve only one spatial dimension, which requires judicious choice of slice and the readout direction. Also, it is not always possible to avoid smaller overlapping vessels that are present in the lung but, from our observations using PC-MRI, through-plane blood flow in these vessels is negligible compared to the ascending and descending aorta in the CD intensity images. Lastly, we reason that 10 ms temporal resolution is sufficient because we can extract 6–8 data points along the upslope of the systole which lasts about 60–80 ms. Because the upslope during this period is approximately linear, estimation of the separation of the wave-fronts has been shown to be robust even at the considerably lower temporal resolution of 25–30 ms [43].

Further studies are needed to assess the reliability of the method but the work represents an initial step toward establishment of quantitative surrogate metrics for assessing vascular reactivity in the central and peripheral vasculature. Thus, the new technique has potential to provide quantitative information for clinical follow-up of individuals with PAD after drug or exercise intervention.

## Competing interests

The authors’ declare that they have no competing interests.

## Authors’ contributions

ML, EM, TF and FW conceived the study. ML, EE, and ZR analyzed and interpreted data. ML and CL worked on the development of the MRI protocol. ML drafted the manuscript. All authors revised the manuscript critically for important intellectual content, read and approved the final manuscript.

## Acknowledgements

NIH RC 1HL099861 and NIH K25 HL111422.
